# Alterations in pectoralis muscle cell characteristics after radiation of the human breast *in situ*

**DOI:** 10.1093/jrr/rrz067

**Published:** 2019-10-28

**Authors:** Christoph Wallner, Marius Drysch, Stephan A Hahn, Mustafa Becerikli, Fleming Puscz, Johannes Maximilian Wagner, Maxi Sacher, Alexander Sogorski, Mehran Dadras, Marcus Lehnhardt, Björn Behr

**Affiliations:** 1 Department of Plastic Surgery, BG University Hospital Bergmannsheil, Ruhr University Bochum, Bürkle-de-la-Camp Platz 1, 44789 Bochum, Germany; 2 Department of Molecular Gastrointestinal Oncology, Ruhr-University Bochum, Universitätsstraße 150, 44780 Bochum, Germany

**Keywords:** irradiation, breast, cancer, muscle

## Abstract

The life-time risk of being diagnosed with breast cancer is ~12%, hence breast cancer is by far the most common cancer among women. The multimodal treatment concept of breast cancer often intends radiation. The utilized ionizing radiation leads changes in the tissue resulting in tissue damage due to an alteration of molecular factors. The goal of this study was to identify the role of muscle-catabolic proteins after radiation of human pectoralis major muscles *in situ*. Tissue of the pectoralis major muscle was collected in 12 breast cancer patients after radiation (maximum 3 years after radiation) undergoing a deep inferior epigastric perforator free-flap breast reconstruction. At the same time, an intraindividual comparison to rectus abdominis muscle was carried out upon free-flap elevation. Immunological properties, cell proliferation, differentiation as well as the expression profile of the muscle tissue were investigated through immunohistological reactions, a DNA-microarray and histology. We found significantly increased neutrophil immigration in the radiated muscle tissue. At the same time, proteins responsible for muscular atrophy and apoptosis were significantly elevated in immunohistochemistry. A DNA microarray detected immunological upregulation and myo-differentiative disorders in radiated muscle tissue. This novel study investigating catabolism in radiated muscle *in situ* can serve as a basis for the treatment of radiation-accompanied muscle disorders.

## INTRODUCTION

The life-time risk of being diagnosed with breast cancer is ~12%, hence breast cancer is by far the most common cancer among women. With an incidence of over 266 120 (15% of all new cancer cases in the USA), there is a relatively high 5-year survival of 89% [1]. Yet, due to its high prevalence, 40000 people die each year from breast cancer in the USA. Looking at the significance of this disease, there are currently more than 187 000 PubMed entries on breast cancer therapy. In the literature, newer therapy methods are emerging, especially in the field of biologics. Nonetheless, adjuvant radiotherapy is a cornerstone of treatment in breast-conserving therapy [2,3]. Additionally, in patients with positive axillary lymph nodes, thoracic radiation is recommended after breast ablation. Postoperative radiation can halve the risk of recurrence and help to increase survival time [2,3].

Although the ideal radiation dose is the subject of clinical debates, the currently most widely used therapy involves a dose of 50 Gy divided into 25 fractions [2,3]. In addition, hypofractionated dosage and boosting with 10–16 Gy reduces recurrence rates [2,3].

Side effects of radiation may occur up to 6 months after completion. Naturally, special attention is paid to the skin. In addition to scarring, pain, pigmentation, skin defects, dryness and blistering can also occur.

Radiation effects on muscle tissue, which lies directly beneath the glandular tissue, were scientifically largely neglected. To our knowledge, there is no literature that demonstrates the impact of intravital ionizing radiation on skeletal muscle tissue differentiation and catabolism at molecular biological levels. Hence, this study aims to characterize the influence of ionizing radiation on muscle catabolism, atrophy and differentiation *in situ*.

Muscle-based breast reconstruction, such as transverse rectus abdominis muscle flap or latissimus dorsi flap, has additional clinical relevance in studies related to muscle radiation, since these muscles are shown to be negatively affected by radiation upon reconstruction. For instance, after radiation there is a higher tendency to scarring, asymmetry and volume loss in these flaps [4]. Nevertheless, autologous reconstruction with e.g. muscle-based flaps is the most reliable option for breast reconstruction with planned radiation [5,6]. The clinical relevance of radiated muscle is also clear with other malignancy related muscle tissue radiation such as sarcoma. Besides surgical tumor excision, adjuvant radiation is a cornerstone of therapy for local control [7]. Not only is surrounding muscle tissue affected by radiation but also potential muscle-based flap reconstruction for tissue defects after surgical tumor excision.

Some factors play a crucial role in the catabolism and apoptosis of muscle cells [8]. Regarding factors orchestrating muscle cell alterations due to radiation, only a few are described as yet. The MAPK/ERK pathway may play a central role in muscle cell reaction to radiation [9]. So far, however, ‘classic’ catabolic muscle proteins like GDF8 or GDF11 are not described to be involved in radiated-muscle catabolism. We present a novel study comparing radiated and non-radiated human muscle intraindividually.

The goal of this study is to identify the role of muscle-catabolic proteins after radiation of muscle *in situ*. Additionally, we want to explore the potential chronic inflammatory response of muscle tissue to radiation.

## MATERIALS AND METHODS

### Selection of subjects

Tissue harvest and experiments were performed prospectively in accordance and with the approval of the ethical committee of the Ruhr University Bochum (Clinical Trial approval number: 16-5932-BR). Female patient were included in the study who suffered malignant breast cancer over the last 3 years followed by radiation. Informed consent was obtained from female patients between the ages of 30 and 55 without any acute or chronic muscular disease, infection, malignant (except malignant breast cancer) or autoimmune disease. After acquisition of informed consent, a muscle biopsy (size ~0.5 cm^3^) was collected during a microsurgical breast reconstruction from the radiated pectoralis muscle, and an intraindividual control from the rectus abdominis muscle, while preparing these tissues for the microsurgical procedure. Tissue biopsies were directly either frozen to −80 °C or fixed in paraformaldehyde.

### Tissue preparation and histological procedures

The harvested muscle was fixed in 4% paraformaldehyde for 20–24 h, then hydrated, embedded in paraffin and cut into 6–8 μm serial sections. Tissue samples were stained with haematoxylin and eosin (H/E) for analysis of tissue damage and neutrophil infiltration. For immunohistochemical stainings of cleaved caspase-3 (rabbit, polyclonal IgG; SantaCruz Biotechnology, sc-7148, 1:100, Caspase-3(H-277)), GDF-8 (rabbit, polycloncal, SantaCruz Biotechnology, sc-6885-R, 1:50, GDF-8), GSK3-beta (3D10) (mouse, monoclonal, CellSignaling, 9832S, 1:50, GSK3-beta), MYOG (mouse, monoclonal, SantaCruz Biotechnology, sc-377460, 1:50, MYOG (G-1)), FBXO32 (mouse, monoclonal, Santa Cruz Biotechnology, sc-166806, 1:100 MAFbx (F-9)) slides were incubated at 70°C for 60 min. Then, slides were deparaffinized, rehydrated and subsequently incubated with 0.125% Proteinase K at 37°C for 15 min. After a short washing step in PBS, sections were treated with blocking serum for 30 min, washed again in PBS and afterwards incubated with primary antibody diluted in blocking solution overnight at 4°C. After washing with PBS, a rabbit or goat secondary antibody conjugated with AlexaFluor594 or mouse IgG kappa binding protein (SantaCruz Biotechnology, sc-516179, 1:2000, m-IgGκ BP-CFL 647) was used for detection. Afterwards the sections were stained with 4′,6-diamidino-2-phenylindole (DAPI) and subsequently mounted with Fluoromount Aqueous Mounting Medium (Sigma Aldrich). Images for immunofluorescence were taken with a fluorescence microscope (Olympus IX3-Series). In order to analyze stainings, four regions of interest per section were chosen (1000 x 1000 pixels). By using the Adobe Magic Wand Tool (settings: tolerance 60%; noncontiguous) immunohistochemical positive-stained pixels were selected automatically and divided by countable nuclei. Afterwards a mean value was calculated.

For measuring the infiltration of neutrophils four regions of interest per section (stained with H/E) were chosen (1000 × 1000 pixels) and neutrophils counted by three independent persons. For the morphometric analysis, an unbiased sampling procedure was applied. The fraction of normal muscle cells was calculated by measuring the cross-sectional diameters of fibers in four regions of interest in transverse sections (stained with H/E). Cells with a diameter within 10% of the mean control group values were considered ‘normal cells’. Percentage of normal cells was calculated accordingly.

For evaluation of fibrotic tissue Masson-Goldner stainings were performed as described before [10]. Analogous to the H/E stainings, as described above, four regions of interest per section were chosen (1500 × 1500 pixels) and pixel selection was performend semi-automatically by utilizing the Adobe Magic Wand Tool (settings: tolerance 60%; noncontiguous). Thereafter a mean value was calculated.

### RNA microarray

RNA was directly isolated from whole snap-frozen muscle biopsies without any additional pre-selection of cells using the RNeasy Fibrous Tissue Mini Kit (Qiagen, Hilden, Germany) according to the manufacturer’s protocol. RNA quality was assessed via NanoDrop. A 260/280 ratio ≥2 and 260/230 ratio of 1.8–2.2 were considered sufficient for further analysis.

An amount of 100 ng of every total RNA sample was hybridized to an individual Agilent whole genome expression microarray (Human GE 4x44K, v2 G4845A, AMADID 026652, Agilent Technologies) according to the Agilent single color protocol. RNA labeling, hybridization and washing were carried out according to the manufacturer’s instructions. Images of hybridized microarrays were acquired with a DNA microarray scanner (Agilent G2505B) and features were extracted using the Agilent Feature Extraction image analysis software (AFE) version A.10.7.3.1 with default protocols and settings. The AFE algorithm generates a single intensity measure for each feature, referred to as the total gene signal (TGS), which was used for further data analyses using the GeneSpring GX software package version 14.9.1. AFE-TGS values were normalized by the quantile method. Subsequently, data were filtered on normalized expression values. The gene expression data from our study have been deposited in the NCBI’s Gene Expression Omnibus (GEO) database (accession number GSE118380).

### Statistical analysis

Results of the study are presented as mean ± standard error of the mean (SEM) of at least three independent experiments. In order to test the normal distribution of all parameters we have performed the Kolmogorov-Smirnov goodness of fit test. Then, *P*-values were calculated by using Student’s t-test comparing two groups and ANOVA if comparing more than two groups. Statistical significances were set at a *P*-value < 0.05.

For the identification of differentially expressed genes, only entities where at least two out of the total number of samples had values within the selected cut-off (50th–100th percentile) were further included in the data analysis process. Using the GeneSpring GX software package version 14.5, pairwise comparisons of filtered and normalized single-color array data were used to identify differentially expressed genes via moderated t-test. The *P*-values were adjusted for multiple testing according to Benjamini and Hochberg [false detection rate (FDR)] and results were considered statistically significant at adjusted *P*-values <0.05. Lastly, only mRNAs with a fold change ≥2.0 in the microarray analyses were further considered.

## RESULTS

### Patient data

Twelve patients were included in the study. The mean age of the patients was 46.6 ± 5.1 years. All included patients received external radiation of 50 Gy over a course of 5–7 weeks. The mean time between diagnosis and breast reconstruction (biopsy) was 1.6 ± 1.0 years; between radiation and biopsy it was 1.3 ± 0.9 years. Threee patients suffered from ductal carcinoma *in situ*, six patients had an invasive lobular carcinoma and three patients had a ductal carcinoma of the breast.

### Exposure to radiation leads to neutrophil infiltration, destruction of muscle fibers and muscle fibrosis

**Fig. 1:**
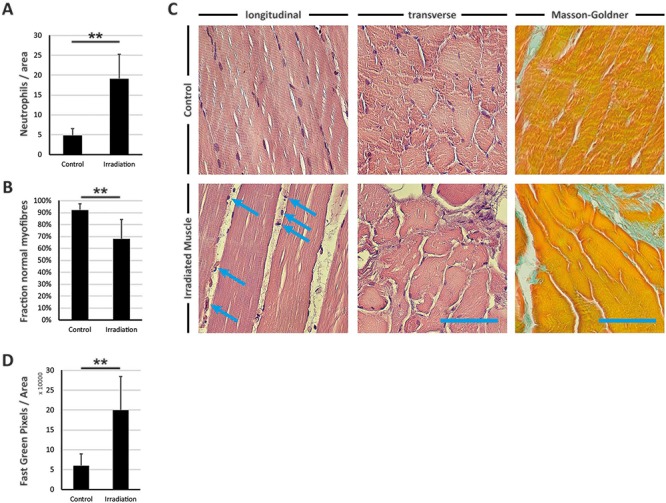
H/E and Masson-Goldner staining of radiated muscle and control muscle from the abdominal wall. (**A**) Illustration of neutrophil infiltration per area (numeric evaluation from (C) longitudinal). (**B**) Illustration of fraction of normal myofibers (numeric evaluation from (C) transverse). (**C**) H/E staining of radiated muscle and control muscle from the abdominal wall in transverse and longitudinal sequence. Blue arrows indicate neutrophils. (**C**) Right-hand column, Masson-Golder staining with Fast Green indicating collagenous tissue. (**D**) Illustration of Fast Green-positive pixels per total area (1500 × 1500 pixels). Results are shown as means ± SEM. Scale bar, 100 μm. ^**^P < 0.01 (two-sample t-test, ANOVA).

We identified an increased neutrophil infiltration into the radiated muscle (3.97 times higher, *P* < 0.01) compared with control tissue of the abdominal rectal muscle. The fraction of normal myofibers in radiated muscle was reduced to 68% compared with the control group with 92% (*P* < 0.01) (see Fig. 1). Moreover, the entire architecture of the muscle was dramatically altered. Masson-Goldner staining of adjacent sections revealed increased Fast Green-positive pixels in radiated muscle (3.3 times higher, *P* < 0.01) compared with control tissue.

### Exposure to radiation leads to reduced expression of BMP2/5, NADPH oxidase 4 (NOX4), OSM and FGF2/9 but higher expression of CXCL1/2/8, CCL21, IL1 and PTGS2

**Fig. 2:**
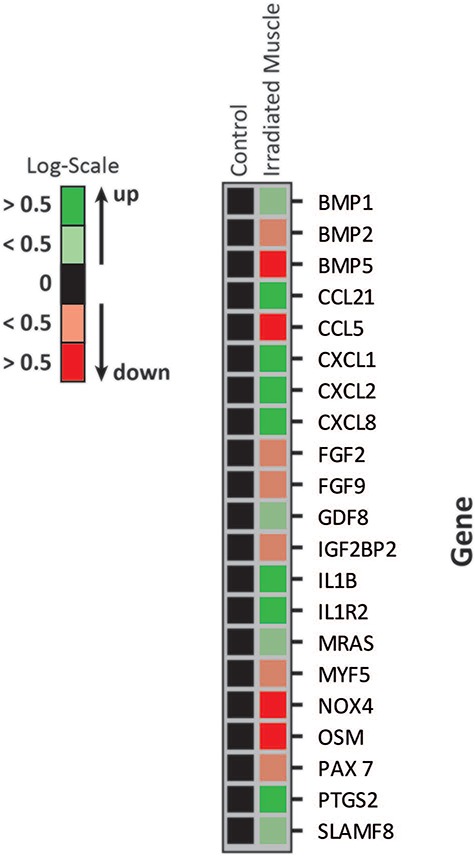
RNA microarray of control muscle and radiated muscle lysates. Gene expression is shown in logarithmic scale with *P* < 0.01. Green indicates an increased expression of at least >0.5, light green indicates an increased expression <0.5, light red indicates a decreased expression <0.5, red indicates a decreased expression >0.5.

Using microarray analysis, we sought to identify altered gene clusters that play a key role in muscle differentiation, inflammation and catabolism. Transcription of growth and differentiation hormones like BMP2/5, FGF2/9, MYF5 and PAX7 was significantly reduced in radiated muscle tissue compared with control tissue from the abdomen. GDF8 transcription was shown to be elevated. Transcription of pro-inflammatory cytokines like CCL21, CXCL1, CXCL2, CXCL8, IL1B, IL1R2, PTGS2 and SLAMF8 was increased in radiated tissue. NOX4 and OSM were highly downregulated in radiated tissue (see Fig. 2).

### Radiated muscle shows higher protein levels for GSK3-β, GDF8, FBXO32 and cleaved CASP3, whereas the level of MYOG protein is decreased

**Fig. 3:**
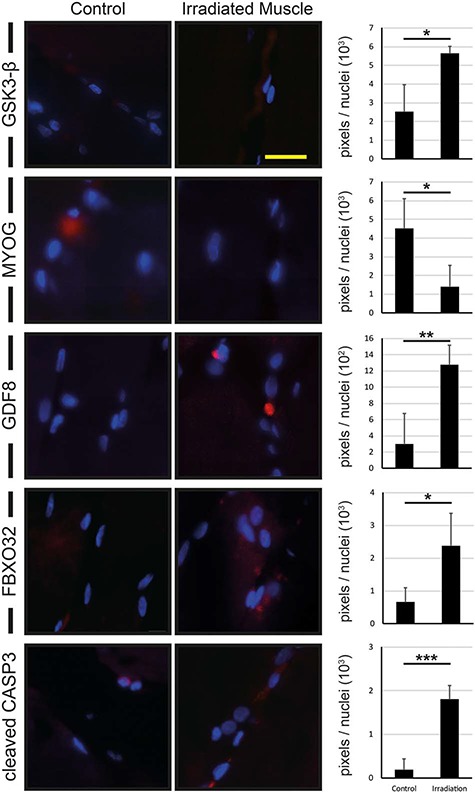
Immunohistological reactions of control muscle and radiated muscle. Immunohistological reactions for GSK3-β, GDF8, FBXO32 and cleaved CASP3 show an increased protein signal in radiated muscle compared with control muscle, whereas MYOG shows a downregulation of the corresponding signal in radiated muscle compared with control muscle. Results are shown as means ± SEM. Scale bar, 40 μm. ^*^P < 0.05, ^**^P < 0.01, ^***^P < 0.001.

To investigate the influence of ionizing radiation on catabolism at protein level, immunofluorescence studies of GSK3-β, GDF8, FBXO32 cleaved CASP3 and MYOG were performed. We were able to show an increase in GSK3-β (indicative of muscle atrophy) protein concentration in radiated muscle by a factor of 2.2 compared with control muscle. MYOG (indicative of myogenesis) level in radiated muscle was reduced to 31% of the level in control tissue. The protein concentration of GDF8 (indicative of muscle atrophy) was elevated by a factor of 4.2 in radiated muscle compared with control tissue. FBXO32 and cleaved CASP3, which is indicative of apoptosis, were increased by factors of 2.3 and 9.4 respectively in radiated muscle compared with control muscle tissue (see Fig. 3).

## DISCUSSION

In this study we revealed that ionizing radiation leads to chronically aberrant expression of genes and correlating proteins of catabolism and inflammation in muscle tissue. As a result, there is increased apoptosis and immune cell infiltration into radiated muscle. We were able to show which proteins and genes are highly up- or down-regulated, thereby identifying specific pathways for potential treatment options. Therefore, this study not only serves as a basis for molecular-biological aspects of radiation damage to breast muscle after breast cancer but also for radiation damage to muscle in general.

There are many studies related to acute effects of ionizing radiation. The current state of knowledge on radiation-induced biological factors includes DNA-damage, cell death and senescence, cellular stress, tumor (neo-)antigens, abscopal effects, hypoxia, immune cell response and soluble factors. Many of those effects are not applicable for the chronic damage after ionizing radiation. Compared with hypoxia-related acute muscle damage, there is less neutrophil infiltration and a higher proportion of normal muscle fibers as found in the current study [11]. Nonetheless, over expression of immune regulation genes such as CXCL1/2/8, CCL21, IL1 and PTGS2 suggests a clear immune response even in the chronic phase after radiation. This is also reflected in the infiltration of neutrophils. A recent study by Pinzur *et al*. showed a systemic upregulation of CXCL1, IL6 and CXCL8 in the radiation of human placental stromal cells *in vivo* [12]. Both our results and the results of Pinzur *et al*. suggest a potent reaction to radiation with elevation of cytokines responsible for white blood cell recruitment and migration. Whereas Pinzur *et al*. demonstrated an acute reaction, we were able to provide evidence of a prolonged upregulation of those cytokines and even neutrophil infiltration. NOX4, a monocyte and neutrophil attractant, was found to be elevated in radiated muscle in our study [13]. SLAMF8, a factor of lymphocyte activation, and OSM, a modulator of the extracellular matrix inducing inflammation, were both elevated in radiated tissue in our study [14,15]. Expression of other radiation specific immunomodulatory cytokines such as IFN-α/β, calreticulin and CXCL12, which were previously shown to be overexpressed in the first 24 h to 7 days, were not found to be significantly altered in our (chronic) study setting. Factors changing tumor microenvironments upon radiation like TGF-β in the form of GDF8 were upregulated as previously shown [16]. On the other hand, many cascades described in acute ionizing radiation like TRAIL, PECAM, VCAM, many other pro-inflammatory cytokines, NFκb, endothelial activation, autophagy, or activation of the inflammasome were not altered in our study [17].

MRAS was found to be moderately elevated in radiated muscle. This factor is expressed in multiple tissue types including muscle. It has an elementary interference with the MAPK-pathway and Akt kinase activity [18,19]. It is also involved in cell cycle arrest that might be associated with decreased myogenic differentiation [20,21].

The combination of reduced expression of FGF 2/9, PAX7, MYF5 but increased expression of GDF8 shows a catabolic, anti-myodifferentiative and anti-myoproliferative environment in radiated muscle [22]. The increased protein concentration of GSK3-β, GDF8, FBXO32 and MYOG in immunofluorescence studies confirms this catabolic and atrophic regulation in radiated muscle tissue [23,24]. Similarly, an increased fraction of cleaved caspase 3 in the radiated muscle suggests muscular atrophy. We were able to demonstrate increased muscular fibrosis in radiated muscle. BMP1 as a factor involved in scarring was elevated in radiated muscle tissue, whereas BMP2 and BMP5 were decreased [25].

Our study suggests a pro-inflammatory, anti-myoproliferative and anti-myodifferentiative environment in the chronic phase following radiation *in situ*. Not only muscle tissue in general but also plastic surgical methods to cover defects with muscular flaps are affected by radiation after cancer. Our results show different factors which play a key role in negative muscle tissue alteration after radiation. This could serve as a basis for future research on treatment options for muscle tissue damage due to ionizing radiation. Bimagrumab is an activin type II receptor antagonist (target of myostatin) intended to be used in patients after hip replacement or patients with general muscle cachexia to reduce muscle catabolism [26,27]. Taking our results into account, the administration of bimagrumab could be used in all patients undergoing radiation with muscle: e.g. breast cancer, lung cancer, prostate cancer, brachytherapy total body irradiation in leukemia. Blockade of the myostatin could also be one possible option to improve the muscular flap quality before radiation in e.g. sarcoma patients or breast patients. Irradiation is also reported to suppress satellite cell function which could potentially be recovered by myostatin blockade [28].

Another important aspect of the study is to increase the awareness of the vulnerability of surrounding tissue of irradiated organs. We have found a large number of deranged proteins even in the chronic state after radiation. While skin damage after radiation is an omnipresent aspect in the precautions and mind of all radiation therapists, long-term damage of muscle as the largest volume organ is still disproportionately under-represented. While the use of myostatin pathway blockers like bimagrumab offer a low side-effect profile, they could serve as a safe treatment to protect muscle tissue before radiation.

## CONFLICT OF INTEREST

We have no actual or potential conflict of interest in relation to this study.

## FUNDING

This work was supported by a grant of FoRUM K108-16 (Research grant by the Ruhr University Bochum, School of Medicine).
